# The role of gut microbiota in the pathogenesis and treatment of postpartum depression

**DOI:** 10.1186/s12991-023-00469-8

**Published:** 2023-09-27

**Authors:** Sheng Zhang, Baili Lu, Gang Wang

**Affiliations:** Wuhan Mental Health Center, Wuhan, China

**Keywords:** Gut microbiota, PPD, Gut-brain axis, Neuroendocrine regulation, Antidepressants

## Abstract

Postpartum depression (PPD) is a common complication of pregnancy in women, and its pathogenesis mainly involves disturbances of the neuroendocrine regulation, immune system, neurotransmitters, hormone secretion, and the gut microbiome. Gut microbes play essential physiological and pathological roles in the gut-brain axis’ pathways which are involved in various central nervous system (CNS) and psychiatric disorders, including PPD. Numerous studies have identified the fundamental role of the gut-brain axis in the pathogenesis and treatment of PPD patients and also correlates with other pathogenic mechanisms of PPD. Disturbances in gut microbes are associated with the disruption of multiple signaling pathways and systems that ultimately lead to PPD development. This review aimed to elucidate the potential connections between gut microbes and the established PPD network, and this might serve as a guide for the development of new efficient diagnostic, therapeutic, and prognostic strategies in the management of PPD.

## Introduction

Postpartum depression (PPD) is one of the common complications of childbirth occurring in 10%–15% of women and is mainly characterized by depression, sadness, frustration, crying, irritability, hallucinations, suicide drives, and a series of symptoms of mental disorders[1, 2]. The Diagnostic and Statistical Manual of Mental Disorders, Fifth Edition, Text Revision (DSM-5-TR) assigns a “postpartum depression” specifier to episodes of depression that begin within six months of delivery. PPD has been variously defined as depression occurring between 4 weeks and 12 months after childbirth [2]. Predictors of PPD include perinatal anxiety, psychiatric illness, life stress, social isolation, low self-esteem, low socioeconomic status, obstetric complications, history of physical abuse, postpartum medical complications, and family history of PPD [3–6]. As many as 70% of pregnant women experience mild depression or depressive episodes, mainly in early and mid-pregnancy [2, 7]. The prevalence of PPD is also influenced by medical and economic conditions, ranging from 10% in high-income countries to more than 20% in some low- or middle-income countries. Additionally, PPD may also be aggregated in families, with women more likely to develop PPD if a sister or mother had the disorder [8, 9].

PPD is not limited to mothers but extends to fathers as supported by a recent meta-analysis of 74 studies which revealed the prevalence of PPD in fathers to be 8.4% [10]. Some of the risk factors for paternal PPD appear to be similar to those for maternal PPD, including primarily financial stress and a past history of depression [10–12]. The natural duration of PPD is variable, with most cases subsiding with treatment within a few months, but 24% and 13% of women diagnosed with PPD remain depressed 1 and 2 years after delivery, respectively [13]. PPD is often distinguished from other complications of childbirth. Shortly after delivery, women usually experience a mild transient syndrome of depressed mood or irritability often referred to as “postpartum blues” or “postpartum psychosis”, which often lasts less than a few weeks and may progress to postpartum depression [14, 15].

In addition, PPD is not only a source of huge economic and medical burden to the society and individuals but also a source of familial conflicts. Children of mothers with PPD often suffer adverse consequences in the long-term [16–18] which among others include decreased feeding, lack of verbal communication and emotional bonding, which can lead to reduced language competence and activity levels, increasing family conflicts and thereby affecting the harmony of the whole family [19]. Moreover, PPD-related suicide has been reported to be the second most common cause of death in postpartum women [20]. There is still a lack of safe and effective therapeutic options, both Traditional Chinese medicine (TCM) and Western medicine, due to the sensitivity of pregnant women to medications. Therefore, the exploration of new treatments for PPD has become extremely urgent. In recent years, gut microbiota has been shown to play an important role in the onset and progression of the central nervous system and psychiatric disorders. Interestingly, PPD occurs mainly after delivery, and dramatic changes in gut microbiota are also major postpartum events [21]. This, together with the fact that gut microbiota plays a key regulatory role in depression progression, lets us speculate that gut microbiota is likely an additional key causative factor in PPD.

There are about 3 million genes of intestinal microorganisms in the human body, which is more than 100 times the number of the human genome [22]. The *Firmicutes* and *Bacteroides* are the most prominent phyla in the human gut and constitute more than 75% of the intestinal microorganisms [23]. The ability of intestinal microorganisms to play a wide range of biological roles depends largely on their quality and quantity. It has been first observed that gut microbiota exhibit differences in terms of microbial composition and abundance between obese and lean individuals [24]. Subsequent studies have gradually highlighted the link between gut microbes and various systemic disorders, including CNS disorders, immune system disorders, and PPD.

The pathogenesis of PPD has not been fully elucidated but it is believed to involve four aspects including brain dysfunction, the hypothalamic–pituitary–adrenal (HPA) axis, immune response, and the brain-gut axis [25]. The causal relationship between gut microbes and mental illness through the gut-brain axis has attracted much attention in recent years. The bidirectional regulation of the gut-brain axis has been investigated for many years and includes the autonomic nervous system (ANS), neuroendocrine system, and metabolism-related pathways, and disturbances in these pathways have been found to be associated with PPD [26, 27]. This is supported by a recent meta-analysis study which showed changes in gut microbiota in patients with depression at phylum and genus levels compared to controls [28], suggesting the important role of gut microbes in the pathophysiology of depression. Differences in gut microbiota are also evident between pregnant women with PPD and those without PPD [29]. In addition to the altered gut flora, the microbial-derived metabolites, including those associated with bile acids and tryptophan metabolism, were also reported to be altered accordingly and thus have an impact on the host organism [30]. Therefore, this implies that intestinal microorganisms play an important role in regulating the homeostatic balance of the PPD.

PPD presents with unique features including altered immune, endocrine, and nervous systems, and epigenetic alterations. Interestingly, changes in any of these systems can alter the homeostasis of microbial composition and structure, suggesting that the gut microbiome might be a pivotal point among the above-mentioned systems [31]. Moreover, several studies reported numerous changes in gut microbes throughout pregnancy [32, 33], hence, gut microbes might serve as a biomarker for PPD. The next sections will highlight the current evidence on the changes in gut microbes in the PPD model and their possible association with other pathogenic factors. Search procedures and study selection Relevant studies were identified by searching Web of Science and PubMed using postpartum depression and gut microbiota as keywords to search terms. Existing meta-analyses, relevant reviews and reference lists of retrieved articles were examined for further relevant studies. DSM-5-TR criteria defined disorders were also used as review disease references. This review belongs to an inductive review, which focuses on the introduction of postpartum depression and gut microbiota and the direct regulatory.

## Gut microbiota and postpartum depression

### Function of normal gut microbiota

A vast amount of research has led to a better understanding of the role of gut microbes in human health and disease development. It is clearly known that metabolic molecules from the gut microbiota can cross the intestinal epithelium into the internal circulatory system to act on the central nervous system. Microbial metabolism produces short-chain fatty acids (SCFA), catecholamines, histamines, gamma-aminobutyric acid (GABA), and other microbial neuromolecules that may directly or indirectly affect brain metabolism and function [34, 35]. Gut microbes are key regulators of the gut-brain axis, and different gut microbes can influence the production of neurotransmitters and precursors such as serotonin, GABA, and tryptophan and regulate the secretion of metabolites including neuropeptides, brain-derived neurotrophic factor (BDNF), and short-chain fatty acids [36–38]. In turn, external stress stimuli can influence the brain to regulate cortisol release and control gut enteric nervous system function and gut mucosal protein production [39, 40]. In addition, the bidirectional signaling of the gut-brain axis not only influences the CNS but also involves both arms of the ANS, including sympathetic and parasympathetic, and regulates the enteric nervous system (ENS) [41]. Therefore, the gut microbiota is a central component of the immune and metabolic system of the host and also can influence the etiopathogenesis of enteric and CNS diseases such as motility, behavioral, and neurodegenerative disorders [42]. The interrelation of gut microbiota and the nervous system via the gut-brain axis suggests that alterations in the gut microbes could be associated with the etiopathogenesis of PPD.

### Changes in gut microbiota and its related metabolites in PPD

Gut microbes are highly associated with molecules related to pathophysiological mechanisms involved in PPD. Recent studies have shown that gut microbes can be involved in multisystem regulation to cause the development of depression-like phenotypes in hosts [43–46]. The PPD phenotype mainly includes anxiety- and depression-like symptoms, and changes in gut microbiota are highly correlated with these phenotypes, suggesting that the gut microbiota is likely to be implicated in the development of PPD after childbirth [47]. Gut microbes are an important regulator of the host homeostasis, and are subjected to drastic changes after delivery. However, when these changes become uncontrollable, they can easily lead to gut microbiota disorders, which, in turn, increase the risk of PPD. Although changes in gut microbiota vary between different PPD models, in general, the variation mainly involves the *Bacteroides*, *Firmicutes,* and *Lactobacillus* phyla [48, 49].

Several studies have reported changes in the gut microbiota in humans and rodents with PPD (Table 1). In a high-fat-diet (HFD)-induced PPD mice model, it was found that at the phylum level, increased abundance of *p_Actinobacteria*, *p_Proteobacteria*,* p_Deferribacteres*,* p_unkunown_Unassigned*, and* p_Verrucomicrobia* was accompanied by decreased abundance of *p_Bacteroidetes*,* p_Cyanobacteria*,* and p_TM7* in PPD mice [29]. At the genus level, increased abundance of *g_Lactobacillus*,* g_Desulfovibrionaceae*, and* g_Christensenellaceae* was accompanied by decreased abundance of *g_unknown_S24-7*,* g_F16*,* and g_Flexispira* compared to the control mice [29]. A similar trend was found in rats in a gestational diabetes mellitus (GDM)-induced PPD model. In this study, an increase in the abundance of *f_Prevotellaceae, f_Ruminococccaceae, f_Peptococciaceae, f_Coriobacteriaceae, g_Prevotella, g_Lactobacillus, and g_Paraprevotella* and a decrease in the abundance of *p_Actinobacteria, p_Clostridiales, f_Lachnospirnaceae, g_Helicobacter, g_Clostridium XlVa, and g_Ruminococcus* was reported in the PPD group compared to the control [50]. In addition to rodents, direct evidence of changes in gut microbiota has been found in human PPD patients. In a study examining 16 healthy and 28 patients with PPD, an increase in the abundance of *p_Actinobacteria, p_Bacteroidetes, and f_Enterobacteriaceae* was found to be accompanied by a decrease in the abundance of *p_Firmicutes, f_Faecalibacterium, f_Phascolarctobacterium, f_Butyricicoccus, f_Lachnospiraceae, g_Faecalibacterium, g_Phascolarctobacterium, g_Butyricicoccus, and g_Megasphaera* in PPD patients [51]. Moreover, prenatal and postnatal stress in rhesus monkeys has been shown to alter the composition and the total number of gut microbes, with the greatest variation in *Lactobacillus* [52, 53]. Interestingly, the ratios of *Firmicutes* and *Bacteroidetes* (F/B) significantly increased in the postpartum depressive subjects in the above-described studies [52, 53]. These changes in the gut microbiota in the depressed subjects provide direct evidence of the relationship between gut microbiota and PPD, suggesting that alterations in gut microbiota might likely play a major role in the development of PPD in both rodents and humans.

**Table 1:** Changes in gut microbiota in different animal models and humans with postpartum depression (PPD)

Experimental subjects	Increased abundance of gut microbiota	Decreased abundance of gut microbiota	References
HFD-induced PPD in C57BL6/J mice	*p_Actinobacteria* *p_Proteobacteria* *p_Deferribacteres* *p_unkunown_Unassigned* *p_Verrucomicrobia* *g_Lactobacillus* *g_Desulfovibrionaceae* *g_Christensenellaceae*	*p_Bacteroidetes* *p_Cyanobacteria* *p_TM7* *g_unknown_S24-7* *g_F16* *g_Flexispira*	[29]
28 patients with PPD and 16 healthy controls (HCs)	*p_Actinobacteria* *p_Bacteroidetes* *f_Enterobacteriaceae*	*p_Firmicutes* *f_Faecalibacterium* *f_Phascolarctobacterium* *f_Butyricicoccus* *f_Lachnospiraceae* *g_Faecalibacterium* *g_Phascolarctobacterium g_Butyricicoccus g_Megasphaera*	[51]
Stress-induced PPD in BALB/c mice	*p_Deferribacteres* *c_Deferribacteres* *o_Deferribacterales* *g_Desulfovibrio g_Alistipes g_Unclassified_Lachnospiraceae, g_Mucispirillum* *s_Eubacterium plexicaudatum, s_Eubacterium sp. 14.2* *s_Lachnospiraceae bacterium 3.1* *s_Mucispirillum schaedleri, s_Desulfovibrio piger*	*f_Rikenellaceae* *g_Turicibacter* *s_Bifidobacterium pseudolongum* *s_uncultured bacterium*	[54]
GDM-induced PPD in SD rats	*f_Prevotellaceae* *f_Ruminococccaceae* *f_Peptococciaceae 1* *f_Coriobacteriaceae* *g_Prevotella* *g_Lactobacillus* *g_Paraprevotella*	*p_Actinobacteria p_Clostridiales* *f_Lachnospirnaceae* *g_Helicobacter g_Clostridium XlVa g_Ruminococcus*	[50]

In different models of PPD, the changes in gut microbiota are accompanied by other molecular changes. For example, in rodent PPD models, perturbations in the microbiome have been associated with a decrease in 5-hydroxytryptamine (5-HT, also known as serotonin), and norepinephrine (NE), and inflammatory response, in combination with cognitive deficits and depressive-like behaviors [29, 50]. However, in human PPD patients, changes in certain gut microbiota have been linearly related to age, weight, body mass index, and sex hormones [55]. The molecular changes in the PPD models are very variable according to the model, but gut flora disorders occur in all the above-mentioned PPD models. From this perspective, it is likely that gut microbiota is probably a major factor in PPD pathogenesis. This is supported by the fact that external interventions, including high-dietary fiber intake or 919 syrup, were able to reverse gut microbiota disorders and their corresponding depressive-like symptoms [29, 54]. It has also been reported that the decrease in the endogenous anti-inflammatory mediators as well as a decrease in the omega-polyunsaturated fatty acids (PUFA), which are observed after parturition, might play a role in the development of PPD [56]. Interestingly, the gut microbiota is central to the host lipid metabolism [57] suggesting that alterations in the gut microbes following child birth could be related to these changes. Moreover, many studies have reported that fecal microbiota transplantation (FMT) can induce or reverse gut microbiota disorders and the accompanying cognitive impairment and depression-like symptoms in the CNS and psychiatric disorders. Varying microbiome levels from phylum to genus are likely to correlate with the microbiome abundance and diversity of the species [58–60]. It is also clear from the above-described studies that gut microbiota might be a critical player in the study of mechanisms related to PPD. Thus, we would further describe the interrelation between gut microbiota and the molecular alterations that occur in PPD in the next section.

## Mechanisms of gut microbiota implication in PPD pathogenesis

**Fig. 1:**
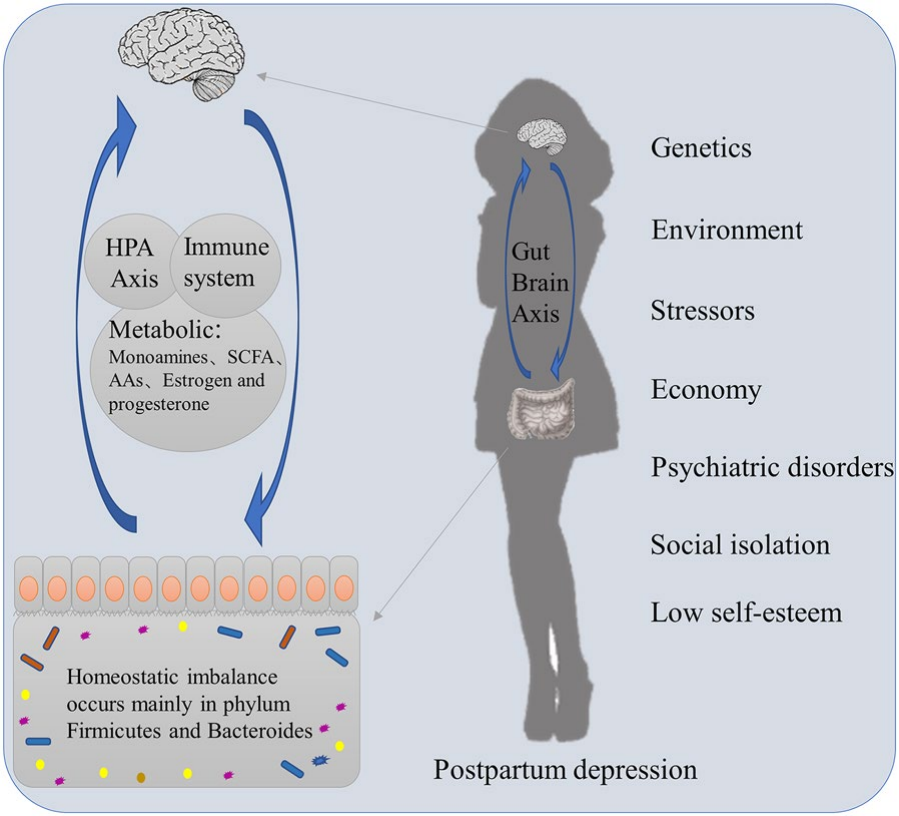
A variety of factors including genes and the external environment can affect the internal HPA axis, immune system, and metabolic disorders through the gut-brain axis and thus contribute to PPD

Also defined as the second brain of the human body, gut microbiota might play a central role in psychiatric disorders, including PPD. Previous studies have demonstrated that alterations in the gut microbiota occur in PPD, and these changes simultaneously modulate multiple systemic molecules including estrogen and progesterone, 5-HT, SCFA, and amino acids (AAs) and the HPA axis to jointly induce PPD (Fig. 1).

### Estrogen and progesterone

Estrogen is a protein produced by the ovaries and placenta that promotes the development of secondary sexual characteristics and the maturation of sexual organs in females. Natural estrogens mainly include estradiol, estrone, and estriol. Oxytocin is a hormone associated with delivery, maternal attachment, and breastfeeding. Mothers with low oxytocin levels during pregnancy show a lower frequency of interaction with their infants, and low maternal oxytocin levels during pregnancy are significantly associated with an increased risk of postpartum pregnancy and susceptibility to PPD development [61]. During pregnancy dynamic, physiological changes affecting the reproductive, immune, nervous, and metabolic systems occur in all pregnant women and these are expected to resolve to a pre-pregnancy state after delivery. Unsuccessful return to normal pre-pregnancy state added to other risk factors has been shown to be associated with the onset of PPD [62]. Therefore, pregnant women undergo dramatic and sudden changes in hormonal levels, such as a decrease in steroid hormones (estradiol and progesterone) following parturition. Studies in this field have provided some evidence that PPD tends to be accompanied by low allopregnanolone and high cortisol concentrations, suggesting that hormonal alterations are closely related to PPD [63, 64]. For instance, the hormone withdrawal theory states that the dramatic postpartum drop in estradiol and progesterone is critical for the development of PPD [65, 66]. Interestingly, the certain gut microbiota is associated with the secretion of β-glucuronidase, an enzyme that breaks down estrogen into its most biologically active form [55, 67] suggesting that gut microbiota might be associated with systemic estrogen and progesterone levels. The disturbance of gut microbiota after delivery might, therefore, cause a dramatic decrease in both estrogens and progesterone by affecting key enzymes (e.g., β-glucuronidase) related to the production of hormones, thus, causing PPD onset. In the postpartum hypoestrogenic state, estrogen production is also a possible mechanism by which the microbiota is involved in PPD onset.

In PPD subjects, fluctuations in estrogen and progesterone levels can trigger changes in the HPA axis, causing women with PPD to be more insensitive to adrenocorticotropin-releasing hormone (CRH) and adrenocorticotropic hormone (ACTH) postpartum, relative to normal women [68, 69]. Sex steroid hormones, such as estradiol and progesterone, can modulate neurotransmitter systems, including the serotoninergic, GABAergic, and dopaminergic systems. In line with this, several studies have shown that an acute decrease in allopregnanolone levels after delivery might promote PPD through GABA receptors [70–72]. Although both estrogen and progesterone are significantly decreased in PPD, administration of both hormones was clinically unsuccessful in reversing PPD symptoms. This is supported by results from a study that showed that women with a history of PPD exhibited increased depressive symptoms after hormone supplementation compared to women without PPD [68]. However, another trial found that estrogen treatment might reduce PPD symptoms, but that synthetic progestin application increased the risk of PPD probably due to the complexity of the body’s biological systems and the fact that progestin supplementation might be influenced by other organismal systems, such as the gut-brain axis [73]. The wide variety and function of intestinal microorganisms can be involved in the estrogen and progesterone disorder of PPD through various biological enzymes.

### Hypothalamic–pituitary–adrenal axis

As a classical pathway, the HPA axis plays a key role in the regulation of cortisol levels through the neuroendocrine system. The activation of the HPA axis stimulates the CRH release from neurosecretory cells in the hypothalamus which in turn stimulates ACTH secretion from the anterior pituitary, which enters the circulation and stimulates the production of glucocorticoids from the adrenal cortex, most notably cortisol [74].

Many researchers have investigated the implication of the HPA axis in depression. For instance, the HPA axis was shown to be activated in response to internal and external stresses causing increased cortisol in depressed subjects [75]. Interestingly, cortisol is the major stress hormone that affects intestinal integrity, motility, and mucus production and might cause changes in the composition of the gut microbiota. Upregulation of this pathway has been reported in depressed patients, ultimately resulting in irritable bowel syndrome and disruption of the gut microbiota [76]. Interestingly, pregnant women with PPD exhibit more pronounced changes in cortisol after social stress [77]. Moreover, women who developed PPD after delivery have elevated CRH levels in mid-pregnancy, and the severity of PPD positively correlates with CRH levels, compared to women who did not develop PPD after delivery, however, ACTH, another protein in the HPA axis, was not correlated with PPD [78, 79].

The gut microbiota is believed to be one of the potential mechanisms regulating the HPA axis via both the activation and inhibition of the HPA axis. Consistently, a study examining the relationship between the HPA axis and stress has found that germ-free (GF) mice showed significantly elevated plasma ACTH and corticosterone levels with a decreased expression of BDNF in the hippocampus and cortex after undergoing restraint stress compared to specific pathogen-free (SPF) mice [40]. Interestingly, the HPA axis activation exhibited by the GF mice could be reversed by *Bifidobacterium* remodeling [40]. In a rat PPD model, the disturbance of gut microbiota was also accompanied by changes in cortisol, suggesting a regulatory role of gut microbiota in the activation of the HPA axis [50]. Together, the data reviewed here provided experimental evidence for the role of gut microbiota in the modulation of PPD through the HPA axis.

### 5-hydroxytryptamine (5-HT)

The discovery in the middle of the last century that the anti-hypertensive reserpine triggered major depression and reduced monoamines raised interest in monoamine neurotransmitters (serotonin, norepinephrine, and dopamine) research. These pathological changes are thought to underlie many neurological and psychiatric disorders, including PPD [80]. Serotonin is one of the most significant neurotransmitters involved in mood generation, and years of clinical research support the central role of 5-HT in the pathophysiology of depression. Consistent with this, a reduced affinity for serotonin and platelet receptors and a reduced action of serotonin in the brains of women with PPD were reported [81]. Moreover, in PPD, a decline in 5-HT production was observed and this is thought to be among the main causes of depressive episodes. In addition to changes in serotonin, tryptophan, the sole precursor of serotonin, is also disturbed during pregnancy [82, 83]. Interestingly, many studies have highlighted the role of gut microbiota in the metabolism of both serotonin and tryptophan.

The regulation of host 5-HT has been reported to be influenced by different gut microbiota. For example, the spore-forming bacteria (Sp) and their specific metabolites were found to promote 5-HT biosynthesis in colonic enterochromaffin cells (ECs) and increase blood, mucosal, and lumen 5-HT levels [84]. In addition, the regulation and production of 5-HT have been reported to be influenced by other microbes including *s*_*Candida, s_Streptococcus,* and *s*_*Escherichia* [85]. Interestingly, it was found that colonic colonization levels and 5-HT levels have been significantly decreased in GF mice, whereas tryptophan hydroxylase 1, the rate-limiting enzyme in 5-HT biosynthesis, was significantly increased in GF mice after microbiota transplantation from normal mice [86], suggesting a pivotal role of commensal gut microbiota in regulating 5-HT homeostasis. In a chronic unpredictable mild stress (CUMS) depression mouse model, *Clostridium butyricum* was found to attenuate depressive-like behavior by increasing 5-HT, Glucagon-like peptide-1 (GLP-1), and Glucagon-like peptide 1 receptor (GLP-1R) concentrations and upregulating BDNF expression [87]. Similarly, *Lactobacillus* was used to examine the alleviating effects in the CUMS depression model, and *Bifidobacterium E41* and *Bifidobacterium breve M2CF22M7* significantly reduced depression-like behaviors in forced swimming and sugar-water preference experiments in mice. Further investigation of the underlying mechanisms revealed that these microbes increased 5-HT and BDNF levels in the mouse brain [88], suggesting that *E41* and *M2CF22M7* exert antidepressant-like effects in depressed mice in a 5-HTP-dependent and microbially regulated manner. In a study examining the effect of gut microbiota and antidepressants, *Bifidobacteria* was found to reduce depression-like phenotypes through the 5-HT reuptake pathway compared to antidepressants [89]. In the HFD-induced PPD mice model, disruption of gut microbiota was accompanied by depressive-like behavior, while high dietary fiber (inulin) intake prevented disruption of gut microbiota and depressive-like behavior in PPD through the microbial-gut-brain axis [29]. Further analysis indicates that an increase in microorganisms such as *Lactobacillus* and *S24-7* as well as 5-HT tryptophan regulation play a major role in the improvement of depression-like symptoms. In another study, FMT intervention on the early gut microbiota of neonatal piglets was able to regulate serotonin and tryptophan metabolism in the host [90], providing a new basis for the regulation of depression-like phenotypes by the gut microbiota through the regulation of tryptophan metabolism.

### Short-chain fatty acids (SCFAs)

Some of the gut microbiota-derived metabolites include SCFAs which are important mediators in the gut-brain axis. SCFAs can reduce chronic inflammation through direct consumption producing local and systemic effects through host cells, or binding to host cell receptors, which is a common marker of PPD [91]. This suggests that the level of SCFA intake and production during pregnancy might also impact the PPD status in pregnant women, indicating the role of SCFAs in PPD.

The depressive symptoms in PPD could be affected by the composition as well as the structure of the individual microbiome through the gut-brain axis. In an HFD-induced mouse model of PPD, high dietary fiber intake prevented gut microbiota disturbances and depression-like behaviors by remodeling gut microbiota and increasing SCFA levels, including acetate, propionate, butyrate, and isovalerate [29]. Also, it was found that in depression patients the production of gut-associated microbial metabolites, such as tryptophan and SCFA, is associated with serotonin metabolism and HPA axis regulation [92, 93]. Consistent with this, it was reported that bacterial end-products such as acetate, propionate, and butyrate, all increase the expression of tyrosine and tryptophan hydroxylases which are involved in the synthesis of dopamine, norepinephrine, and serotonin, and thus, are critical for neurotransmission processes [94–96]. Moreover, it has been identified that gut microbiota can regulate kynurenic acid by affecting tryptophan production, and steroid and glucocorticoid levels by SCFA-mediated stimulation of the serotonin synthesis in the intestinal endothelium [86, 97]. Some microorganisms present in our gut, e.g., *Akkermansia muciniphila and Ruminococcus obeum*, can degrade fiber into SCFA such as acetic acid, propionic acid, and butyric acid [98], which might explain the ameliorative effect of high dietary fiber intake in HFD-induced PPD in mice. Some reports have suggested that butyrate might have antidepressant-like properties [91, 98]. Particularly, sodium butyrate was reported to significantly improve depression-like behaviors in CUMS-induced mice at least in part via increasing brain 5-HT level, BDNF expression, and improving Blood–brain barrier (BBB) impairments [91]. Moreover, sodium butyrate was reported to improve aversive memory impairment, depression-like behaviors, and hippocampal microglial activation via inhibiting histone deacetylase and increasing the levels of Ten-Eleven Translocation 1 (TET1), 5-HT, and BDNF expression [99–101]. Lipophilic SCFAs readily cross the blood–brain barrier to reach the brain and directly interact with neurons. The application of sodium butyrate has restored normal levels of the prefrontal cortex, hippocampus, striatum, and amygdala and reversed depressive- and manic-like behaviors in rats [102]. The above-mentioned evidence indicates that SCFA is an intermediate molecule in the depression-like phenotype and could be modulated by gut microbiota.

### Amino acids

Recently, disruptions in AA in both GDM-induced rat PPD and stress-induced mouse PPD models were reported. In the rat PPD model, a decrease in the ratio of *Firmicutes* to *Bacteroidetes* as well as a decrease in the expression of tryptophan hydroxylase (TPH), a key enzyme in the 5-HT production pathway, resulted in a significant decrease in 5-HT, while in the mouse PPD model, the disruption of the gut microbiota was accompanied by a significant decrease in 4-aminobutyric acid [50, 54]. Meanwhile, blood levels of the AA serine, methionine, asparagine, glutamine, and tryptophan were reported to be reduced in depressed patients, whereas elevated plasma levels of phenylalanine, aspartate, serine, and γ-glutamyl AAs were found in such patients [103, 104]. Importantly, these studies demonstrated that the changes in plasma AA levels correlate with changes in AAs observed with gut microbial metabolism which might be the pathological basis for gut microbial-induced depression. These studies indicate that some of these AAs and their bacterial-derived metabolites might serve as biomarkers of PPD in clinical practice. Glutamate (or glutamine) is another disturbed AAs and their levels are significantly elevated in the brain, cerebrospinal fluid, and plasma of patients with depression [105]. Glutamate is an agonist of the *N*-methyl-d-aspartate (NMDA) receptor, and interestingly, NMDA receptor antagonists such as ketamine have been reported to show rapid antidepressant properties in animal models of depression as well as in depressed patients, by inhibiting glutamatergic neurotransmission [106].

In agreement with the data reviewed above, the metabolic interaction of AAs in the host and the microbes as biomarkers of PPD is present in many gut bacterial species. Such interactions between bacteria and hosts occur at both local and systemic levels. AA metabolites involved in branched-chain AA synthesis, namely leucine, isoleucine, threonine, and methionine, have been shown to be dysregulated in patients with major depressive disorder (MDD) [107]. The main members of the AA-producing gut microbiotas are *s_Streptococcus, Staphylococcus aureus, Escherichia coli, s_Klebsiella, s_Ruminomonas, Ehrlichia megaterium, s_Proteus, s_Synechococcus,* and *s_Clostridium* [108]. These gut microbes can directly influence the onset of PPD through AA production and modulation. Among these AA, alanine, arginine, glutamine, leucine, methionine, serine, tryptophan, and tyrosine have all been shown to exert some varying degrees of antidepressant effects in different experimental systems [109]. The microbe *L. reuteri 3* was reported to increase the expression of serotonin biosynthesis enzymes while inhibiting the expression of enzymes involved in tryptophan metabolism in the kynurenine pathway in the frontal cortex, ultimately improving depression-like behavior [110]. Moreover, clinical studies have revealed that d-serine is one of many AAs with antidepressant effects similar to ketamine, as demonstrated in animal studies conducted in rodent models of depression [111, 112]. Therefore, the data reviewed here strongly suggest that disturbance of gut microbiota as seen in the PPD model as well as in PPD patients could often be reflected by changes in AAs as well suggesting that AAs play a critical role in PPD and could serve as potential biomarkers.

### Other mechanisms

In addition to the above-discussed common pathophysiological mechanisms, many genetic and environmental factors are involved in the etiopathogenesis of PPD. For example, Specific genes encoding female reproductive hormones, stress, inflammation-related components, and neuropeptides were reported to be altered in PPD [113]. Genetic alterations in the serotonin transporter gene (SERT) oxytocin receptor (OXTR) gene, the oxytocin peptide gene, the glucocorticoid receptor gene, and the CRH receptor 1 gene have been associated with PPD [114]. Estrogen-induced DNA methylation is also associated with PPD development, suggesting a genetic component in PPD pathogenesis [115], and DNA methylation biomarkers were revealed to predict both antenatal and postpartum depression [116].

Numerous negative environmental factors can also exacerbate PPD onset. For instance, family misfortune, internal isolation, lack of cultural background, and low social status are all risk factors for PPD. Particularly, studies have highlighted that women who are in economic distress have a 2–3 times higher risk of developing PPD than women who are economically independent [117–119]. Although there are studies exploring the potential mechanisms of the external environment in PPD, more evidence is needed to understand its impact.

The immune system is also an integral part of the pathogenesis of depression. This is supported by the studies that indicate that prolonged HPA axis hyperactivity due to increased proinflammatory cytokines such as IL-1, IL-6, and TNF-α is one of the underlying mechanisms of cytokine-induced depression [120]. Moreover, a recent study also revealed that alteration in B-cell activation and insulin resistance might also be implicated in PPD [121]. Other factors like zinc, vitamin D, high-density lipoprotein (HDL)-cholesterol, and omics biomarker have all been reported to play a role in the development of PPD [114]. A lot of studies on PPD related to genetic, environmental, and other factors are ongoing, and many reviews have been published on the implications of genetic factors in the pathophysiology of PPD [114, 115, 122].

## Treatment of PPD

Current prevention, diagnosis, and treatment strategies around PPD are severely underdeveloped, largely due to limited reporting by patients with PPD and reluctance to seek outside help, care, and pharmacological interventions, especially during breastfeeding. Screening after delivery is a key to early detection and diagnosis of PPD, and clinicians can improve the detection and diagnosis of PPD using a reliable screening tool, such as the Edinburgh Postpartum Depression Scale (EPDS) [123]. Given the specificity of maternal conditions, women with PPD tend to prefer psychotherapy to medication and want to avoid medication, especially while breastfeeding [124]. This occurs in form of counseling in one-on-one or group therapy. Moderate to severe PPD often requires medication administration to have a positive effect, and this usually results in the reduction of gut microbiota disturbances and depressive-like symptoms. The main antidepressants used in clinical practice are serotonin reuptake inhibitors (SSRIs), tricyclic antidepressants, serotonin–norepinephrine reuptake inhibitors (SNRIs), and monoamine oxidase inhibitors [125]. Among them, selective SSRIs have become the first-line drugs. The safety profile of SSRIs in breastfeeding is relatively high, with few reports of maternal and infant adverse effects [126]. SNRIs or mirtazapine are usually administered when SSRIs are ineffective or when these drugs were previously indicated for women, as the available data have suggested that SNRIs are rarely introduced into breast milk [127]. Monoamine oxidase inhibitors are used less frequently because of their potential side effects, and their safety during breastfeeding is also unknown. Unlike the above-preferred medications, tricyclic antidepressants are usually avoided during breastfeeding because of their potential entry into the breast milk and cardiac side effects [128]. Recently, an allopregnanolone-based PPD treatment brexanolone has been approved by the Food and Drug Administration (FDA) in the United States as a fast-acting and long-lasting antidepressant [114]. Widely used antidepressant therapeutic regimens still have a high number of drawbacks, including drug tolerance, delayed action, suboptimal efficacy, and various adverse effects [129]. Therefore, the search for new potential treatments for PPD is significant, and gut microbiota treatment might be a promising option.

Gut microbiota have a wide range of physiological roles, including nutritional delivery, engagement in human metabolism, immunity, regulation of brain function, regulation of bone density, and anticancer. Dysregulation of gut flora is closely associated with many diseases such as autoimmunity, metabolism, neurological disorders and even cancer, such as obesity, diabetes, inflammatory bowel disease, colorectal cancer and Alzheimer's disease [130]. Interestingly, normal microbiota can be restored through probiotics, prebiotics, diet, and fecal microbial transplantation in animal models [131–133]. Metabolite tryptophol acetate produced by probiotic yeast blocks group sensing of several pathogenic gram-negative bacteria, thereby inhibiting bacterial virulence [134]. Several studies in rodent and macaque models of depression have found that transplantation of normal microbiota to depressed animals reversed the host depression-like phenotype [60, 135]. These studies provide experimental evidence for the clinical application of gut microbiota therapy; however, it has not been yet performed in human PPD subjects.

## Conclusion and perspective

PPD is one of the most common complications of childbirth, and if left untreated, it can have far-reaching negative effects on the mother, child, and the whole family. Thus, safer and more effective treatments are urgently needed. Research has revealed that disruptions in the gut microbiota, the “second brain” can lead to the development of multisystem diseases, including PPD. In this study, we reviewed data from both experimental and clinical research that indicate the implication of gut microbiota dysregulation in the pathogenesis of PPD. Direct evidence revealed gut microbiota disorders in patients with PPD, which are mainly clustered in phyla *Firmicute, Bacteroide*, and *Actinobacteria* in PPD. The molecular changes are highly variable in different types of PPD subjects, however, there are always gut microbiota disturbances. The application of gut microbiota remodeling in a PPD model can reverse gut microbiota disorders and their accompanying depressive-like symptoms. This suggests that gut microbiota plays a causative role in the bidirectional communication of the “gut-brain axis”, in which gut microbiota dysregulation might play a key role in PPD pathogenesis through various mechanisms including serotonin, hormones, and the HPA axis modulation.

Although a huge number of articles have been published on the link between gut microbiota and many diseases including depression, most of them are animal studies, especially fecal microbiota transplantation to treat depressive animals. While treatments for depression or postpartum depression have mostly focused on interventions with small clusters of metabolites, such as SSRIs, SNRIs, etc., those aimed at improving the gut microbiota environment for the treatment of depression have mostly focused on probiotics. However, there is still a long way to go in the research field of gut microbiota and its application in PPD patients. For example, the changes in gut microbiota in different PPD populations with disorders are slightly different, and there is no specific gut microbiota or a more convincing change pattern. The application of gut microbiota to clinical treatment will still take a long time to be realized due to the limitations of human experiments and the sensitive condition of pregnant women. Overall, there have been inspiring advances in the study of gut microbiota, and the corresponding specific changes in gut microbiota can be used as biomarkers of PPD risk for diagnosis, treatment, and prognosis. Most current reviews or articles focus on the etiology, pathogenesis, consequences, and treatment approaches of postpartum depression. This review provides a comprehensive and detailed overview of the role played by gut microbiota in the pathology of postpartum depression from the perspective of gut microbiota on postpartum depression. Gut microbiota therapy is a non-invasive and therefore, a more promising treatment modality, providing a desirable blueprint for the future clinical treatment of PPD patients. The development of specific biomarkers, genetic and epigenetic, for early and better diagnosis of PPD is a prerequisite for management of women with PPD.

## Data Availability

This manuscript, including all relevant raw data, will be freely available to any researcher wishing to use them for non-commercial purposes, without breaching participant confidentiality.

## Abbreviations

AAs: Amino acids
ACTH: Adrenocorticotropic hormone
ANS: Autonomic nervous system
BDNF: Brain-derived neurotrophic factor
CNS: Central nervous system
CRH: Adrenocorticotropin-releasing hormone
CUMS: Chronic unpredictable mild stress
ECs: Enterochromaffin cells
ENS: Enteric nervous system
EPDS: Edinburgh Postpartum Depression Scale
FDA: Food and Drug Administration
FMT: Fecal microbiota transplantation
GABA: Gamma-aminobutyric acid
GDM: Gestational diabetes mellitus
GF: Germ free
GLP: Glucagon-like peptide-1
HFD: High-fat-diet
HPA: Hypothalamic–pituitary–adrenal
MDD: Major depressive disorder
NMDA: *N*-Methyl-d-aspartate
OXTR: Oxytocin receptor
PPD: Postpartum depression
PPP: Postpartum psychosis
PUFA: Omega-polyunsaturated fatty acids
SCFA: Short-chain fatty acids
SERT: Serotonin transporter gene
SNRIs: Serotonin-norepinephrine reuptake inhibitors
SPF: Specific pathogen free
SSRIs: Serotonin reuptake inhibitors
TET-1: Ten–Eleven Translocation 1
TPH: Tryptophan hydroxylase
5-HT: 5-Hydroxytryptamine
